# Never Too Late: Safety and Efficacy of Deep TMS for Late-Life Depression

**DOI:** 10.3390/jcm13030816

**Published:** 2024-01-31

**Authors:** Yiftach Roth, Faisal Munasifi, Steven A. Harvey, Geoffrey Grammer, Colleen A. Hanlon, Aron Tendler

**Affiliations:** 1 BrainsWay Ltd., Jerusalem 9777518, Israel; yiftah@brainsway.com (Y.R.); colleen.hanlon@brainsway.com (C.A.H.); 2 Department of Life Sciences, Ben Gurion University, Beer Sheba 84990, Israel; 3 Tallahassee Brain Stimulation Center, LLC, 1407 MD Lane, Tallahassee, FL 32308, USA; fmunasifi@mdlane.net; 4 Greenbrook TMS Neurohealth, 16091 Swingley Ridge Rd. Suite 100, Chesterfield, MO 63017, USA; sharvey@greenbrooktms.com; 5 Greenbrook TMS Neurohealth, 8405 Greensboro Dr #120, McLean, VA 22102, USA; ggrammer@greenbrooktms.com; 6 DTMS Center LLC, 1601 Forum Place, West Palm Beach, FL 33401, USA

**Keywords:** Deep TMS, rTMS, H-coil, late-life depression, phase IV study, major depressive disorder

## Abstract

Repetitive transcranial magnetic stimulation (rTMS) is an effective and well-established treatment for major depressive disorder (MDD). Deep TMS utilizes specially designed H-Coils to stimulate the deep and broad cerebral regions associated with the reward system. The improved depth penetration of Deep TMS may be particularly important in late-life patients who often experience brain atrophy. The aim of this phase IV open-label study was to evaluate the safety and efficacy of Deep TMS in patients with late-life MDD. Data were collected from 247 patients with MDD aged 60–91 at 16 sites who had received at least 20 Deep TMS sessions for MDD. The outcome measures included self-assessment questionnaires (Patient Health Questionnaire-9 (PHQ-9), Beck Depression Inventory-II (BDI-II)) and clinician-based scales (21-item Hamilton Depression Rating Scale (HDRS-21)). Following 30 sessions of Deep TMS, there was a 79.4% response and 60.3% remission rate on the most rated scale. The outcomes on the PHQ-9 were similar (76.6% response and 54.7% remission rate). The highest remission and response rates were observed with the HDRS physician-rated scale after 30 sessions (89% response and a 78% remission rate). After 20 sessions, there was a 73% response and 73% remission rate on the HDRS. Consistent with prior studies, the median onset of response was 14 sessions (20 days). The median onset of remission was 15 sessions (23 days). The treatment was well tolerated, with no reported serious adverse events. These high response and remission rates in patients with treatment-resistant late-life depression suggest that Deep TMS is a safe, well-tolerated and effective treatment for this expanded age range of older adults.

## 1. Introduction

By 2030, there will likely be over 1.4 billion individuals that are over the age of 60, many of whom will suffer from late-life depression [1]. Major depressive disorder (MDD) is the main cause of disability worldwide [2] and some types of MDD may pose a higher risk for dementia in older adults [3]. An evolving list of pharmacological interventions has been the primary treatment tool for MDD for over 50 years. Yet, about 30% of patients with MDD do not respond well to pharmacotherapy [4,5], including adults with late-life depression [6]. Between 55% and 81% of late-life patients with MDD do not respond to first-line antidepressant treatment [7]. Various studies have found that depression resistant to antidepressants occurs at a rate of 26 to 41 cases per 100 persons among late-life patients suffering from MDD [8,9,10]. Late-life patients with MDD often suffer from comorbid medical disorders and are treated with various medications. As a result, these patients are often at a greater risk of adverse events due to drug–drug interactions [11]. A meta-analysis of randomized controlled trials (RCTs) found antidepressants to be much less efficacious in older patients, with the number needed to treat (NNT) increasing with age, from 6 in studies of general adult MDD, to 8 in patients aged 55 years and above, and to 21 in those older than 65 years old [12]. There is clearly a great need for additional therapeutic options for patients suffering from treatment-resistant late-life depression, and this need is expected to increase in the coming years and decades.

Repetitive transcranial magnetic stimulation (rTMS) is a safe, non-invasive neuromodulation technique. TMS is performed by passing a transient electric current pulse through a coil placed on the scalp. This current generates an electric field pulse in the underlying brain tissue that induces neural depolarization and activation [13]. The repetitive application of TMS (rTMS) may induce long-term neuroplastic changes in the excitability and connectivity of relevant brain circuits. This is believed to underlie its use as a therapeutic intervention for various neuropsychiatric indications [14], including MDD [15,16]. While the application of high-frequency rTMS over the left dorsolateral prefrontal cortex is a widely used therapeutic tool for treating middle-aged adults with MDD, there is less evidence regarding the efficacy of TMS on MDD in patients over 60 years old. While a recent meta-analysis demonstrated high levels of efficacy in this population [17] and a retrospective study found that rTMS has a similar efficacy in patients with MDD above and below 60 years old [18], more data from large samples are needed. 

The goal of this phase IV study was to collect real-world post-marketing surveillance data and outcomes from patients with late-life depression who were treated with the Deep TMS H1 Coil. Deep TMS utilizes specially designed H-Coils to induce neuronal depolarization in deep and broad cerebral regions [19,20]. Deep TMS was FDA cleared for MDD in 2013 following a multicenter randomized sham-controlled trial [21]. Since then, it has been adopted into the continuum of care, with real-world evidence demonstrating high efficacy [22]. 

While Deep TMS is routinely given to adults throughout the life span, there has been some theoretical concern that older adults may not respond as well, given age-related atrophy. This is because age-related atrophy leads to an increased scalp–cortex distance [23]. This results in a lower magnetic field strength in the targeted region of the cortex. That said, two studies recently demonstrated its high efficacy for treating MDD in older populations. In one sham-controlled randomized clinical trial, Deep TMS for MDD in older adults found 40% remission and 44% response rates, which were significantly better than the sham [24]. That study used a larger number of pulses per session, namely 6012, which is three times the dose typically given for MDD. Another study of Deep TMS in patients with MDD 70 years and older found significant improvements in depressive symptoms [25]. Here, we present the results of yet another, larger study that investigated H1-coil Deep TMS at the standard FDA-cleared protocol of 1800 pulses at 18 Hz in an elderly MDD population.

## 2. Materials and Methods

This phase IV study was designed to collect treatment information, demographic data, and outcome data on late-life subjects treated with the Deep TMS H1 Coil for MDD. All Deep TMS clinics across the US were asked to participate and were sent instructions. The subjects’ depression severity was assessed by the 21-item Hamilton Depression Rating Scale (HDRS-21) [26], the Patient Health Questionnaire-9 (PHQ-9) [27] and the Beck Depression Inventory-II (BDI-II) [28]. To incentivize participation and support the work of data entry, clinics received USD 5 per line of data and USD 70 per HDRS assessment. A line of data corresponded to one treatment session with detailed treatment information. All sites received device training and certification. The protocol was reviewed by Sterling IRB and granted exemption from informed consent, provided the patients were assigned only a patient code (no name/initials) and age (year, not date of birth). 

Data were collected between 2018 and 2021 from 16 clinical sites. All sites used uniform criteria and included patients aged 60 years or above seeking treatment for acute depression, allowing co-morbidity from DSM-V. No formal diagnostic assessment was conducted. Patients were screened with the Clinical TMS Adult Safety Screening Questionnaire (TASS). Patients were treated using the BrainsWay H1 Coil with a Magstim Rapid^2^ (Magstim Company, Spring Gardens, UK) stimulator or with the BrainsWay 104 stimulator (BrainsWay, Jerusalem, Israel). For each subject, the individual resting motor threshold (rMT) of the right hand was determined using visual twitches. The coil was then moved 6 cm antreriorly as in the FDA-cleared protocol [21]. Deep TMS was administered according to the FDA-cleared protocol: 18 Hz frequency, 120% intensity related to the hand rMT, 55 trains of 2 s duration, inter-train interval (ITI) of 20 s, and 1980 pulses over 20 min per session. The patients generally received 5 sessions per week. The analyses included the remission (defined as HDRS-21 < 10; PHQ-9 < 5; BDI-II < 13) and response (defined as ≥50% improvement from baseline) rates for patients who received at least 20 and at least 30 Deep TMS sessions for each of the scales and for the scale most rated for each individual. Additional analyses included the median and inter-quartile intervals of the number of sessions and the days required to reach response and remission, as well as the Kaplan–Meier survival curve of response (time to event [response]), which employed the scale most used for each subject. The correlation between age and response and the remission rates were assessed with Pearson’s test.

## 3. Results

**Figure 1:**
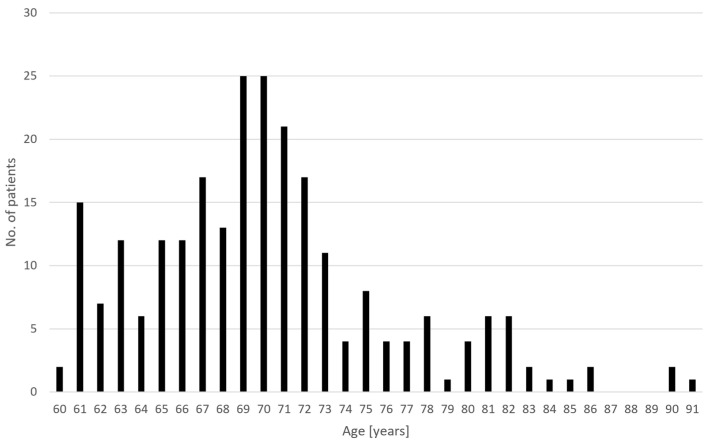
Age distribution of the patients recruited in this study.

The data of 247 patients with treatment-resistant MDD who were between 60–91 years old (mean ± SD = 70.2 ± 6.1) were collected from 16 clinical sites. The patients were 62% female, 96% white, had a current episode with a duration of 21.5 ± 21.5 months and had a history of 8.5 ± 5.1 depressive episodes, receiving 12 ± 5 concomitant antidepressant medications. The baseline PHQ-9 was 18.6 ± 5.2 (mean ± SD). The age distribution is plotted in Figure 1. 

**Figure 2:**
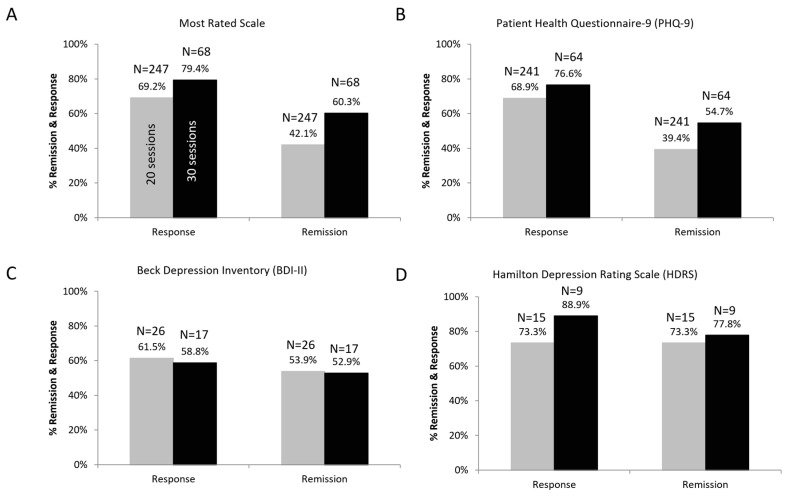
Remission and response rates based on individuals that received 20 sessions (gray) or 30 sessions (black) of Deep TMS with the H1 coil. (**A**) Aggregate data of the most rated scale used for each patient. Subsets of data for the PHQ-9 (**B**), BDI-II (**C**) and HDRS (**D**) are also shown. The number of patients with available data at each interval is presented beside each data point.

The treatment was well tolerated, with no reported serious adverse events. A significant improvement was seen post treatment. The patients’ PHQ-9 scores after 20 sessions were significantly better compared to baseline (*p* < 0.0001; paired *t*-test). Age had no effect on the clinical outcomes. Patients who received 20 sessions had 69.2% response rates and 42.1% remission rates on their most rated scale. This increased to a 79.4% response rate and 60.3% remission rate for patients who received 30 sessions of Deep TMS (Figure 2). Among the 241 patients assessed with PHQ-9, 20 Deep TMS sessions led to a 68.9% response rate and 39.4% remission rate. Treatment with 30 sessions of Deep TMS led to a 76.6% response rate and 54.7% remission rate. Among the 26 patients assessed with BDI-II, 20 Deep TMS sessions led to a 61.5% response rate and 53.9% remission rate. Treatment with 30 sessions of Deep TMS led to a 58.8% response rate and 52.9% remission rate. Remission and response rates were highest among the 15 patients who were assessed with the physician-rated HDRS (73% response and remission rates for 20 sessions; 89% response and 78% remission rates for 30 sessions).

**Figure 3:**
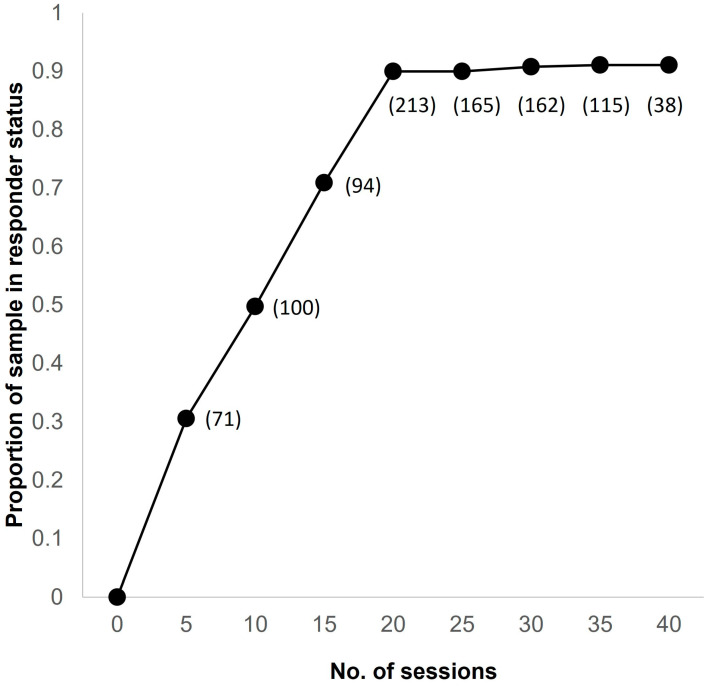
Cumulative incidence (1 survival) plot of response. The event is the first occurrence of ≥50% improvement from baseline in the most rated score among subjects monitored at a given number of sessions. The number of patients monitored at a certain number of sessions is shown next to the data points.

The median [interquartile interval] onset of response occurred after 14 sessions [9,20], which was equivalent to a median of 20 days [12,29]. Remission was achieved after a median of 15 sessions [10,22], which was equivalent to a median of 23 days [15,30]. Kaplan–Meier survival analysis revealed that after 20 sessions, the rate of response was 90% among monitored patients (Figure 3). The number of patients with available data at each timepoint appears next to the data point. 

## 4. Discussion

The results of this phase IV Deep TMS study in late-life MDD extend our existing knowledge regarding the efficacy of this treatment in adults aged 60–91 years old. Of the 247 patients with late-life depression, the response rates, remission rates, and the median numbers of sessions/days required to reach response/remission are comparable to the results of a recent large naturalistic study of Deep TMS in middle-aged adults [22]. Specifically, the response rate was approximately 70% after 20 sessions and 80% after 30 sessions. The remission rate was approximately 40% after 20 sessions and 60% after 30 sessions. The median onset of response occurred after 14 Deep TMS sessions and remission was achieved after a median of 15 sessions. The results demonstrate that the efficacy of Deep TMS is similar in middle-aged patients and those with MDD who are later in life (60–91 years old). 

The response and remission rates were much higher with the physician-based HDRS scale than with the patient self-reported rating scales (PHQ-9 and BDI-II). This finding is in accord with previous studies [22,29,31] and may indicate that patients with MDD are later to acknowledge their improvement than the physicians. Yet, this result should be interpreted with caution since the majority of patients were informed by PHQ-9, while only 15 patients were informed by HDRS-21.

The response and remission rates in this study support and extend previously published controlled and open-label studies of TMS in late-life MDD (see [32] for review). The higher rates of response and remission demonstrated in this study may be a result of a higher dose being delivered here than in previous studies [32], of the larger sample size, and/or of the deeper and broader brain volume directly stimulated with Deep TMS [33]. Trevizol et al. [34] found a 0% remission rate following high-frequency (HF) rTMS to the left DLPFC in patients with MDD aged 60 and older, but a 40% remission rate following bilateral rTMS (HF rTMS to the left DLPFC, low-frequency (1 Hz) rTMS to the right DLPFC). Another larger study by the same group [35] demonstrated that bilateral rTMS and bilateral theta burst (TBS) had remission rates of 32.9% and 35.4%, respectively [35], which are slightly lower than the remission rates seen in the current Deep TMS study (42.1% after 20 sessions, 60.3% after 30 sessions) when calculating the most rated scale. This is similar to a previous Deep TMS RCT, where 20 Deep TMS sessions led to 40% remission [24]. Notably, the response and remission rates are much higher on the physician rated HDRS-21. The H1 Coil produces an electric field with the bilateral stimulation of the prefrontal cortex, with a stronger field over the left hemisphere. It is possible that the simultaneous bilateral stimulation of the PFC via the H1 coil (left and right prefrontal cortex at the same time) with a high frequency (rather than sequentially with facilitatory protocol to the left DLPFC and inhibitory protocol to the right DLPFC) represents another way of optimizing TMS treatment for MDD, as well as for late-life MDD. Future studies should address the question of optimized protocols for older patients suffering from MDD, and whether they should be different from protocols for the general MDD population. In general, the accumulated evidence obtained during rTMS studies in adult and older patients suffering from depression seems to indicate that these interventions are similarly effective in the two populations.

Age-related atrophy leads to an increased scalp–cortex distance in depressed patients, more prominently in the prefrontal cortex compared to the motor cortex [23]. Several studies have shown that a larger scalp-to-cortex distance in the frontal cortex is related to a lower clinical outcome in rTMS treatment for MDD in older adults [36]. No adjustment of the stimulation intensity according to the scalp-to-cortex distance was performed in the current study. Future studies are required to investigate the potential of such adjustment to further improve the clinical outcomes of Deep TMS in late-life patients with MDD. 

The first-line treatments for MDD include various types and combinations of antidepressant medications and/or psychotherapy. Yet, a substantial portion of the MDD patient population does not reach response, even following various medication trials [5] and combinations of pharmacotherapy and psychotherapy [37]. Furthermore, many patients fail to receive adequate doses of medications due to adverse effects [38]. In contrast, Deep TMS treatment is in general very well tolerated, with mild and transient side effects. The most serious adverse event reported is seizures, which are very rare and occur at a frequency of less than 0.001 [39]. The most common adverse events of Deep TMS are headache, treatment site discomfort and facial muscle pain [40]. Most of the adverse events are transient, are resolved within days, and their severity is mild to moderate. Deep TMS, as an add-on to antidepressant medications, has been used in clinical trials (i.e., [41]) and in real-life practice [22]. No interactions that may lead to adverse effects have been reported, and the antidepressant efficacy is similar to Deep TMS as a monotherapy.

Several recent studies have pointed to a potential advantage of using very accurate fMRI-guided neuronavigation TMS with a figure-8 coil, applying an accelerated intermittent theta-bursts (iTBS) protocol to individualized DLPFC targets to ameliorate MDD symptoms [30,42]. Deep TMS represents a different approach that stimulates a broad and deep volume in the prefrontal cortex. A recent post-marketing study found that accelerated iTBS with Deep TMS with no neuronavigation led to very high remission and response rates [43]. It is possible that for focal rTMS, which is performed with a figure-8 coil, there is an advantage in accurate individualized neuronavigation, as there is in the SAINT protocol [30,42]; meanwhile, with the broad volume stimulated by H-coils in Deep TMS, there is lower probability of “missing the target”, and accurate neuronavigation is hence not required. Future studies will have to address these fascinating questions and aid in optimizing TMS treatments for MDD.

The primary limitations of this study are related to its open-label naturalistic design. As such, there may theoretically be various sources of bias. One potential bias is a desire to include the data of patients with better clinical outcomes. Yet, the sites were asked to send all data on patients aged 60 and above seeking treatment for MDD, including comorbidities. The clinics were compensated for every line of data, irrespective of the results. Therefore, the sites were motivated to send as much data as they could, and thus the risk of bias towards better results seems small. Yet, the data received are a small part of all patients treated with Deep TMS. The data were received from 16 sites across the US, but it cannot be guaranteed that the results thoroughly represent the general population of treated patients. In addition, the study is vulnerable to variability in provider practices, environmental variables in the clinics that are difficult to account for in a statistical analysis, and incomplete data sets from some of the patients. A further potential source of bias is that the data do not account for concurrent psychotherapy and medications that may have been initiated along with Deep TMS but not fully reported in this dataset. Another limitation is that long-term follow-up was not available. While the aforementioned concerns are common to phase IV studies, the impact of these concerns on the interpretation of the results is mitigated in part by the consistency observed when comparing the results of this study to middle-aged individuals receiving Deep TMS, as well as the compatibility of the results with other studies that have delivered TMS to individuals with late-life depression. 

## 5. Conclusions

In summary, Deep TMS is demonstrated to be a safe and effective treatment for treatment-resistant patients suffering from late-life MDD who do not benefit from pharmacotherapy and psychotherapy. This study of 247 Patients with MDD aged 60 and above found very high response and remission rates (70% (80%) response rates and 40% (60%) remission rates after 20 and 30 Deep TMS sessions, respectively) and the fast onset of a clinical effect, with a median of 14–15 sessions. These results are comparable to those achieved in a younger population and indicate that Deep TMS is a safe and effective therapeutic option for late-life MDD. 

## Data Availability

BrainsWay routinely shares data with researchers around the world, contact research@brainsway.com.
